# Dermatophytosis Incognito Mimicking Cutaneous T-Cell Lymphoma

**DOI:** 10.7759/cureus.25809

**Published:** 2022-06-10

**Authors:** Shahab Babakoohi, Chad M McCall

**Affiliations:** 1 Oncologic Dermatology, Atrium Health Wake Forest Baptist Medical Center, Charlotte, USA; 2 Pathology, Carolinas Pathology Group, Charlotte, USA

**Keywords:** eczema, terbinafine, ctcl, dermatophytosis incognito, mycosis fungiodes

## Abstract

Dermatophytosis incognito can be missed in diagnosis, given its relatively low prevalence as compared with common cases of dermatophytosis and therefore, is likely under-reported. Cutaneous T-cell lymphoma (CTCL) is also a rare entity with variable clinical manifestations. While successful treatment of dermatophytosis is feasible withmultiple topical and systemic antifungal options, CTCL can present a therapeutic challenge associated with significant emotional distress for the patients. We present a case of tinea incognito initially treated for eczema, later considered biopsy-supported CTCL that was successfully treated with antifungal therapy.

## Introduction

Dermatophytosis incognito is a condition seen in an untreated fungal infection following the application of topical corticosteroids which may not always manifest with typical tinea symptoms [1]. It is often inadvertently treated as inflammatory dermatoses such as eczema prior to the correct diagnosis. Cutaneous T-cell lymphoma (CTCL) includes a broad spectrum of subtypes and clinical manifestations of cutaneous malignant clonal T lymphocytes. Mycosis fungoides is the most common type [2]. CTCL can also be misdiagnosed and treated as inflammatory dermatoses. Despite significant diagnostic advances including cultures, special stains, and molecular pathology in the diagnosis of these entities, clinical suspicion still plays a major role.

## Case presentation

**Figure 1:**
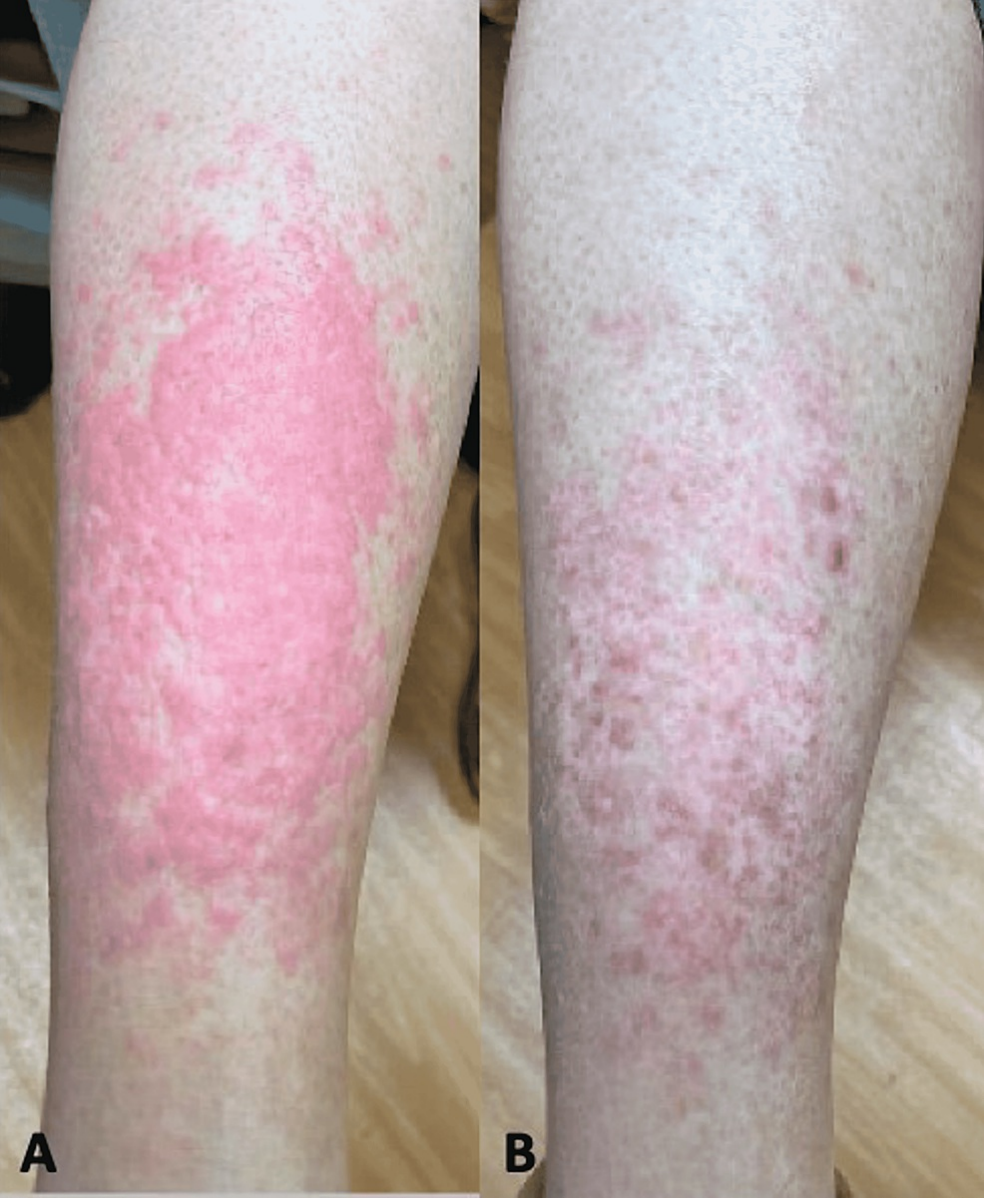
A. Right shin eruption at presentation; B. After one month of terbinafine

The patient was a 62-year-old woman with a history of chronic myeloid leukemia and polycythemia vera with myelofibrosis diagnosed in 2009; she went into remission after a hematopoietic stem cell transplant in 2015. In 2020, she developed a pruritic rash on the right shin with a large plaque and a small rash on the right elbow (Figure 1). She was diagnosed with eczema and treated with multiple lines of potent topical corticosteroids for greater than six months. The rash, inflammation, and pruritus progressively worsened. She was referred to oncologic dermatology.

With a clinical impression of dermatophytosis incognito, empiric ketoconazole cream was started. Systemic treatment was deferred pending the results of pathology, microbiology tissue cultures, and evaluation of response to topical therapy.

**Figure 2:**
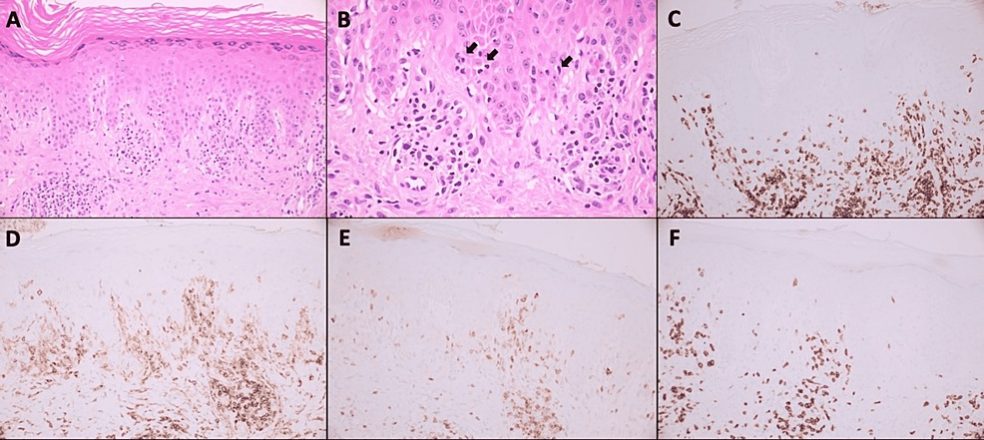
A. Dermal-based lymphoid infiltrate with focal evidence of epidermotropism (H&E, 200x). B. Epidermal lymphocytes with irregular nuclear contours and clear cytoplasm (arrows) (H&E, 400x). C. CD3 immunohistochemical stain highlighting T-cells, which account for most of the lymphoid infiltrate (200x). D. CD4 immunohistochemical stain highlighting the majority of the T-cells, as well as dimly staining dendritic cells and macrophages (200x). E. CD7 immunohistochemical stain showing partial loss of expression in the T-cells (200x). F. CD8 immunohistochemical stain showing background cytotoxic T-cells, fewer in number than CD4-positive cells (200x).

Histopathology of the punch biopsy from the right shin showed an atypical lymphoid infiltrate with small lymphocytes with irregular nuclear contours and clear cytoplasm, which was centered in the dermis with focal evidence of epidermotropism (Figure 2). The infiltrate extended around the adnexal structures and blood vessels and had focal involvement of the hair follicle. A periodic acid-Schiff (PAS) stain was negative for fungal elements. The atypical lymphocytes were positive for CD3 and CD4 with partial loss of CD7 expression. CD8 highlighted background T cells. CD30 was positive in very rare cells. CD20 highlighted very rare background B cells. The findings were compatible with a diagnosis of mycosis fungoides. 

A preliminary discussion was performed with the patient for potential treatment options including both topical and systemic CTCL therapies given the failure of topical corticosteroid treatment. Given the extensive history of hematologic malignancies with complications of systemic treatments, she was unwilling to proceed with systemic agents in case of failure of topical options.

During ongoing discussions regarding CTCL treatment, the patient reported a notable improvement in inflammation of rash after several days of ketoconazole cream application, thrice daily. Prior to the consideration of mycosis fungoides-directed therapy, an oral terbinafine course was tried. T-cell receptor gamma gene rearrangement assay returned negative for monoclonality while receiving oral antifungal agent.

The patient reported significant improvement one week after treatment. The right elbow rash resolved after two weeks of treatment. One month after treatment, the right shin plaque had dramatically improved.

Tissue culture for bacterial, fungal, and atypical mycobacterial infection remained negative. The patient did not return for further follow-up as she reported complete resolution. 

## Discussion

Dermatophytosis incognito is seen in untreated superficial fungal infections treated with corticosteroids. PAS staining sensitivity varies in the literature from 70% to 98% [3]. A negative result does not confidently exclude the diagnosis. There are few studies on the sensitivity of tissue culture for dermatophytes. In one study, clinical assessment was used as the gold standard for tinea pedis. The sensitivities for KOH smear and culture were approximately 73% and 42%, respectively [4].

CTCL, a lymphoma that is generally known as a liquid tumor, has a predilection for the skin. While it is frequently limited to the skin, it can spread into the lymphatic system and even enter the leukemic phase known as Sezary syndrome [5]. CTCL is diagnosed by histopathology features, immunohistochemistry, and molecular markers. In histopathology, diffuse dermal infiltrate of neoplastic T-cells is seen [6]. Among immunophenotypical characteristics, the most common finding is loss of CD7 expression; however, CD7 expression loss can be a reactive phenomenon and has been reported in inflammatory benign dermatosis [7]. Accumulation of CD7 negative T-cell lymphocytes has been reported in lymphocytic infiltrates and inflammatory skin lesions [8] Our patient did regularly shave her legs which is a known risk factor for dermatophytosis infection in the setting of immunosuppression. Her right elbow rash could be an autoeczematization reaction which resolved simultaneously with the improvement of leg eruption.

## Conclusions

Mycosis fungoides and dermatophytosis incognito can have overlapping features. Histopathology, special stains, or culture may not clearly differentiate these two entities. Clinicians need to use their clinical skills in the diagnosis of complex dermatoses, especially when some treatment options would be prolonged, aggressive, or bear considerable side effects.
